# On-farm harvest timing effects on alfalfa weevil across the Intermountain West region of the United States

**DOI:** 10.3389/finsc.2024.1324044

**Published:** 2024-04-23

**Authors:** Judith S. Herreid, Tatyana A. Rand, Darren M. Cockrell, Frank B. Peairs, Randa Jabbour

**Affiliations:** ^1^ Department of Plant Sciences, University of Wyoming, Laramie, WY, United States; ^2^ Pest Management Research Unit, United States Department of Agriculture – Agricultural Research Service (USDA-ARS), Sidney, MT, United States; ^3^ School of Global Environmental Sustainability, Colorado State University, Fort Collins, CO, United States; ^4^ Department of Agricultural Biology, Colorado State University, Fort Collins, CO, United States

**Keywords:** alfalfa hay, physical control, integrated pest management, *Hypera postica*, *Medicago sativa*, *Bathyplectes curculionis*, forage crop

## Abstract

Alfalfa (*Medicago sativa* L.) is an economically important commodity in the Intermountain Western United States. A major concern for alfalfa producers in this region is the alfalfa weevil (*Hypera postica* Gyllenhal). Insecticide resistance development coupled with regulatory changes in pesticide use has resulted in renewed interest by producers in non-chemical control methods such as cultural control. One such cultural control method is early harvest, which consists of producers timing their harvests early in the season to decrease alfalfa weevil damage. This method is thought to be effective by exposing weevil larvae to adverse conditions before significant damage occurs. Still, early harvest can be difficult to employ because recommendations are often vague. To better understand how early harvest impacts both alfalfa weevils and their natural enemies and how producers are using this method across the Intermountain Western United States, we conducted a study in alfalfa production fields in Colorado, Montana, and Wyoming over three growing seasons. We determined that the timing of the initial alfalfa harvest spanned more than 1 month across fields, and alfalfa plant stage at harvest ranged from late vegetative to early bloom. Harvest was more impactful on reducing alfalfa weevil densities the earlier it was implemented. Removing windrows in a timely manner is likely useful to further decrease alfalfa weevil densities. Harvest timing was not associated with parasitism rates of alfalfa weevil, but higher parasitism rates were associated with lower post-harvest alfalfa weevil densities. This work has increased our understanding of early harvest in an on-farm setting and to improve recommendations for producers across the Intermountain Western United States.

## 1 Introduction

Alfalfa (*Medicago sativa* L.) is an important perennial forage crop on both national and global scales. This productive legume provides high-quality livestock feed (1). In recent years, the economic value of alfalfa has substantially increased, especially in the western United States (2). Insect pest control is a major challenge in this system. Agricultural intensification and climate change have the potential to negatively impact farming regions in part through increased pest survival (3). Within the Intermountain Western United States, the alfalfa weevil, *Hypera postica* (Gyllenhal) (Coleoptera: Curculionidae), is of particular concern to growers (4). Alfalfa weevils reduce both yield and quality through plant defoliation, delay plant growth, and cause economic damage (5, 6). The chemical arsenal available to producers to combat alfalfa weevil has diminished over the years due to increased insecticide regulations and the development of pesticide resistance selection (7).

In the western United States, pyrethroid insecticide use has been the primary method of alfalfa weevil control, making the rise of resistance in alfalfa weevil worrisome. Over the past decade, reports of alfalfa weevil pyrethroid resistance have been sporadic across the western United States and Canada (6–8). Recent work demonstrated that although resistance is not found everywhere in the western United States, it is strongly established across the region (7). Thus, cultural methods of control, such as early harvest, can be useful in mitigating alfalfa weevil damage while also managing resistance development (7).

Taking the first harvest before weevils have completed their lifecycle and caused substantial damage can be beneficial for producers (9). Although this method has been recommended for many years, it can be complicated to implement (10). Alfalfa yield and quality generally have an inverse relationship to one another. Early-growth alfalfa is associated with high quality but lower yields, while more mature alfalfa has higher yields but lower quality (11). Consequently, a critical part of early harvest is the determination of the precise timing of harvest while considering the yield/quality trade-off. Harvesting too early could result in inadequate yield and reduce alfalfa plant fitness. Timing the initial harvest too late may result in low-quality alfalfa and inadequate weevil control, causing damage in subsequent harvests (12). Earlier first harvests have the potential to target early instar alfalfa weevil larvae when they are likely more susceptible to mortality. Recommendations are often vague on when early harvest should take place and do not account for important variables related to plant biology and climate (10). Some early work used alfalfa plant height, weevil density, or weevil stage to provide harvest timing recommendations but did not consider yield/quality trade-offs (12–14). Updated recommendations from Onstad and Shoemaker (12) factored in these trade-offs but used growing degree days (GDDs). Alfalfa and weevils may develop at different rates across wide geographic areas, which complicates the use of GDDs as a reliable predictor (15, 16). Additionally, if the goal is to maximize the non-chemical control of alfalfa weevil, determining how natural enemies like parasitoids also interact with harvest timing is critical.

Alfalfa weevil biocontrol in the United States has a long history with parasitoid introductions beginning as early as the 1910s. One of the most widely released biocontrol agents, *Bathyplectes curculionis* (Thomson) (Hymenoptera: Ichneumonidae), was established and dispersed rapidly but does not seem to adequately manage weevil populations on its own (17). Currently, alfalfa weevil biocontrol is moderately effective in some regions but not in the western United States (5, 18). *Bathyplectes curculionis* are generally present in the field when early harvest is being implemented (19). By understanding how their activity impacts alfalfa weevil control or if early harvest could be timed in a way that minimally impacts *B. curculionis*, we may be able to optimize early harvest and maximize non-chemical alfalfa weevil control.

This work examined the first alfalfa harvest of the season on working farms across a wide geographic range in the Intermountain Western United States. We recorded when producers harvested and how this differed based on location. This study also measured the efficacy of harvest for weevil reduction across various harvest timings. Finally, we quantified how activity of the parasitoid *B. curculionis* varied across producer fields and states, and in relation to harvest timing. The overall goal of this work is to increase our understanding of early harvest in an on-farm setting and build better recommendations for producers across large geographic regions that may help slow the development of insecticide resistance.

## 2 Materials and methods

### 2.1 Study location and design

Alfalfa production fields were sampled for three summers (2019, 2021, and 2022) across the Intermountain West in Montana, Wyoming, and Colorado. Field work was not completed in 2020 due to travel and work restrictions related to the COVID-19 pandemic. Teams in each state located producer collaborators by reaching out to previous producer connections and networking with various government and extension professionals to locate additional interested producers. Alfalfa fields were selected based on the following outlined criteria and with producer input. Fields were irrigated, insecticide-free within the year until sampling efforts concluded, and at least 2 km away from one another to ensure spatial independence of landscapes. In total, across 3 years, 10 fields from Montana (or within 10 miles of the Montana state border), 17 fields from Wyoming, and 7 fields from Colorado met these criteria and were included in analyses. Collection fields are not representative of their entire state; within each state, fields were grouped into regions based on geographic separation (**Supplementary Figure 1**). Montana fields were located near the eastern part of the state, with regional groups separated by major rivers in the region (**Supplementary Table 1**). Wyoming fields were located around southeastern Wyoming and grouped into regions separated by non-farming regions (**Supplementary Table 1**). Colorado fields were located east of the Front Range in Colorado, with urban areas separating their regional groups (**Supplementary Table 1**).

To evaluate the impact of harvest on alfalfa weevil, we targeted sampling to collect data in the week prior to harvest and up to 2 weeks following harvest. Communication with producers helped determine when pre-harvest sampling should begin based on when harvest would likely happen, as producers determined their own harvest times for this project. We analyzed data collected from 1 week pre-harvest and 2 weeks post-harvest unless otherwise specified. Generally, harvest takes place between mid-May and mid-June in the Intermountain West. This often coincides with when alfalfa weevil larvae are highly damaging, which is likely due to a combination of later instar alfalfa weevil larvae inflicting substantial feeding injury and alfalfa weevil densities around this time (20, 21). This study design allowed us to determine how harvest is impacting alfalfa weevils when they are most problematic. In each field, both pre- and post-harvest data were collected along a single transect set 30.5 m into the field to mitigate edge effects.

### 2.2 Pre-harvest sampling

In each field, sampling took place at 0.09-m^2^ (1-ft^2^) quadrats 15 m apart at five locations along a single 61-m transect. The specific transect location within a field was chosen randomly. Stem density, height, alfalfa plant stage, damage score, alfalfa weevil density, and alfalfa weevil egg counts were quantified for each of the five locations along the transect within a field. At each quadrat, alfalfa height, stage, and damage were quantified visually. Plant stage determination followed Fick and Mueller (22). Damage scores ranged from 0 (no damage) to 5 (complete defoliation) and are based on the damage scale from Berberet and McNew (23). Within each quadrat, alfalfa was removed with a handheld harvest knife while leaning over a canvas sheet. Alfalfa weevils were collected with a modified shake bucket method (24). Stems were then placed into a large plastic bag, and weevils collected from the shake bucket, on the canvas, and from the ground within the quadrat were placed into a separate plastic bag. To determine the parasitism rate, three transects of 50 sweeps each were taken perpendicular to the 30.5-m collection transect. Each sweep sample was placed in a plastic bag for transport. If weevil densities were too low to provide 50 larvae from three sweep collections, more samples were collected. All collected stem, bucket, and sweep samples were then transported to the laboratory for processing.

In the laboratory, stem density was determined, and 25 stems from each quadrat were split to determine the alfalfa weevil egg count. Stems were then examined for the remaining alfalfa weevils. Weevils collected during this time were combined with the field-collected alfalfa weevils to estimate total weevil density per 0.09 m^2^ (1 ft^2^). They were counted and staged as adult, pupae, or larval instar (1–4) based on chaetotaxy (25). For analysis, larvae were grouped into early instar larvae (sum of first and second instars) and late instar larvae (sum of third and fourth instars). If there were more than 30 alfalfa weevil larvae in a sample, we employed subsampling procedures. First, the total alfalfa weevil larvae count was recorded. Then, a large 14-cm Petri dish was gridded into four equal quadrants. Alfalfa weevil larvae were emptied into the dish and were evenly spread across the dish. Weevils in the top right quadrant were staged up to 20. If less than 20 weevils were in the quadrant, staging proceeded to the next quadrant in a clockwise direction. Staging continued until a minimum of 20 larvae were staged. The alfalfa weevil parasitism rate was determined by randomly selecting 50 late-stage (third and fourth instar) alfalfa weevil larvae from sweep collections to dissect under a microscope and examine for parasitoid larvae.

### 2.3 Post-harvest sampling

Similar to pre-harvest, sampling took place along a 61-m transect at five locations. Instead of a single 0.09-m^2^ (1-ft^2^) quadrat being sampled at each of the five locations, either one or two quadrats were sampled for each location based on whether windrows were present and the team conducting the sampling. Windrows are the gathered cut plant material that is left to dry on the ground before baling, and there is evidence to suggest that alfalfa weevils can use windrows as a refuge to increase their survivorship (26). Regardless of the number of samples collected, when windrows were present, we equally sampled under and adjacent to windrows. A vacuum sampler (leaf blower with reverse suction flow and a net placed inside, as in 27) was used at each quadrat to collect from the ground and vegetation. Vacuum sampling is one of the most effective methods of collecting alfalfa weevils from a harvested alfalfa field with no mature vegetation. Vacuum samples were transported to the laboratory and used to determine alfalfa weevil counts from each quadrat. Additionally, we recorded if windrows had been removed during sampling and if the sample was collected from below or adjacent to a windrow for inclusion in our statistical models.

### 2.4 Data analysis

All statistical analyses were conducted with R 4.2.2 project software in R studio (28, 29). Producer management trends were characterized through summary statistics. Analyses of variance were employed to examine how these management trends vary between Colorado, Wyoming, and Montana. Two negative binomial generalized linear models with log links were conducted to determine how post-harvest and pre-harvest weevil densities were related to harvest Julian day. GDDs were not used as the response variable due to a lack of field-specific weather data needed to calculate this particular metric. Model predictions and 95% confidence intervals were estimated using the “ciTools” package (30). These were examined to determine how weevil densities pre- and post-harvest compare and predict our response harvest Julian day. The earliest harvested field was not included in these analyses. This harvest took place over a week before the next earliest harvest, and its removal improved both models’ performance as it had an outsized impact on model fit. This field was included in subsequent analyses unless otherwise noted. Assumptions of linearity, normality, and homoskedasticity were assessed with diagnostic plots.

A generalized linear model with a quasibinomial error distribution and logit link was run to determine the impact of timing and location on alfalfa weevil instar proportions before harvest. Model predictors, which included harvest Julian day, alfalfa stage, and state, were tested for collinearity. The initial global model included all predictors as well as all possible two- and three-way interactions with early instar alfalfa weevil proportion as the response. The early instar alfalfa weevil proportion is calculated by taking the total number of early (first and second) instar alfalfa weevil larvae in a collection and dividing it by the total number of all alfalfa weevils at any life stage in that collection. We focused on the early instar alfalfa weevil because that cohort, in particular, is targeted by early harvest practices. All non-significant interactions were removed from the model. All model assumptions were met.

To examine what impacted post-harvest alfalfa weevil densities, we used field location information and pre- and post-harvest collected data to create a set of generalized linear mixed models (GLMMs). A negative binomial error distribution with a log link function was employed for all models, and the “glmmTMB” package was used for our GLMMs with random effects (31). Post-harvest weevil density from week 2 was our model response variable. Our starting global model included fields nested within geographic regions as a random effect. Fixed effects included pre-harvest weevil density, early instar alfalfa weevil proportion, alfalfa stage, alfalfa damage score, alfalfa stem density, harvest Julian day, alfalfa weevil egg count, windrow status (removed from field vs. remaining on field), state, and year. Fixed effects were examined for collinearity with visualizations and variance inflation factor; the only correlation observed was between alfalfa height and alfalfa damage. Taller alfalfa was correlated with lower levels of alfalfa weevil damage. Alfalfa weevil damage is known to reduce alfalfa growth, which explains this correlation (21). We removed alfalfa height from the analysis because of this correlation. Although alfalfa height could be used as a way to inform producers when to harvest, plant height can be impacted by a variety of factors we did not account for (e.g., weather or alfalfa variety) (32, 33). Our global GLMM was simplified with backwards selection to optimize AIC. Predictors for the final model included Harvest Julian day, windrow status, pre-harvest alfalfa damage, state, year, alfalfa weevil egg count, and early instar larvae proportions. Assumptions were checked throughout modeling with the DHARMa package (34).

Finally, to examine parasitoid activity during the first alfalfa harvest and the potential impact on alfalfa weevil, we used two different generalized linear models. We tested the effect of harvest Julian day, pre-harvest weevil density, and the proportion of early instar larvae on our response, and determined the *B. curculionis* parasitism rate prior to harvest using a quasibinomial generalized linear model with a logit link. We also examined parasitism rate as a predictor of post-harvest weevil density using a gamma error distribution with a log link. The post-harvest density metric used was the median number of weevils collected per 0.09-m^2^ (1-ft^2^) quadrat in each field within a week after harvest. This was based on all the subplot weevil collections in each field since parasitism rate was a per-field metric. The median was used instead of the mean because it is a metric that does not give outliers the same influence as the mean, and outliers were present in our weevil counts. These analyses only included fields from Montana and Wyoming because parasitism rate was not collected the week prior to harvest for the fields in Colorado.

## 3 Results

### 3.1 Producer management trends

**Figure 1:**
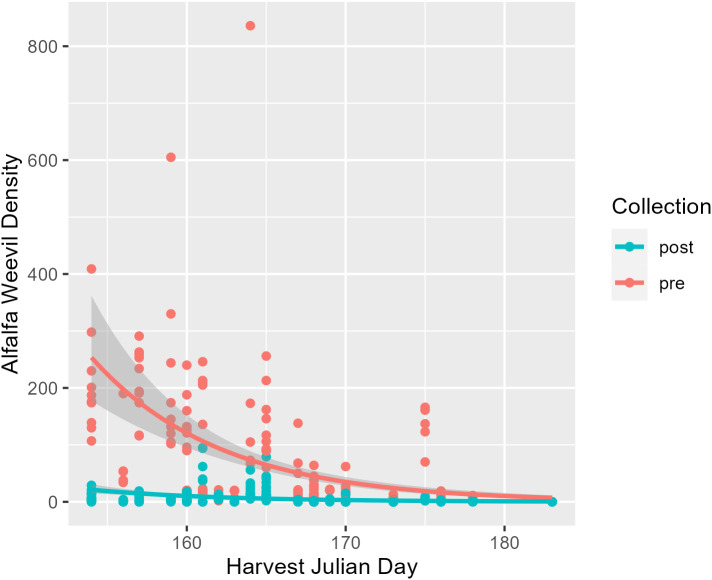
Pre-harvest (red) and post-harvest (blue) alfalfa weevil counts plotted against harvest Julian day with model predictions and their 95% confidence intervals. Each point represents a single 0.09-m^2^ (1-ft^2^) subplot so there are multiple points per unique field.

The first harvest date was recorded for 34 field collections across the Intermountain West where the harvest Julian day ranged from 145 to 183 (**Table 1**). This corresponds to just over a 1-month period starting at the end of May until early July. The average and median initial harvest day across all fields were both Julian day 165, which falls in mid-June (**Table 1**). Alfalfa plant stage the week before harvest ranged from stage 2 (late vegetative) to stage 6 (late flower). On average, alfalfa was at a 3.6 stage before harvest, which falls between the early and late bud stages (**Table 1**). Harvest Julian day did not vary between states (*p* = 0.07; **Appendix 1**; **Supplementary Figure 2**), and neither did alfalfa plant stage at harvest (*p* = 0.36; **Appendix 1**; **Supplementary Figure 3**). Harvest Julian day was related to both alfalfa weevil pre- and post-harvest densities (**Figure 1**). Pre-harvest and post-harvest weevil densities were lower at sites with later harvest dates (**Appendix 1**; pre-harvest: *p* ≤ 0.001; post-harvest: *p* ≤ 0.001). Differences between predicted alfalfa weevil densities and their 95% confidence intervals were larger at earlier harvest Julian days compared to later harvest Julian days (see **Supplementary Table 2**).

**Table 1:** Management trends observed for fields in Colorado, Montana, and Wyoming.

	Harvest Julian Average (σ)	Harvest Julian Median (IQR*)	Harvest Julian Range	Harvest Plant Stage Average (σ)	Harvest Plant Stage Range
Colorado	162.6 (± 9.0)	163.0 (± 7.5)	145–173	3.8 (± 0.6)	3.0–4.6
Montana	169.7 (± 4.3)	169.0 (± 4.3)	162–178	3.4 (± 0.6)	2.8–4.0
Wyoming	163.1 (± 8.2)	160.0 (± 8.0)	154–183	3.7 (± 0.7)	3.0–5.2
All fields	164.9 (± 7.9)	165 (± 9.8)	145–183	3.6 (± 0.6)	2.8–5.2

*IQR = interquartile range.

### 3.2 Alfalfa weevil instar proportions

**Figure 2:**
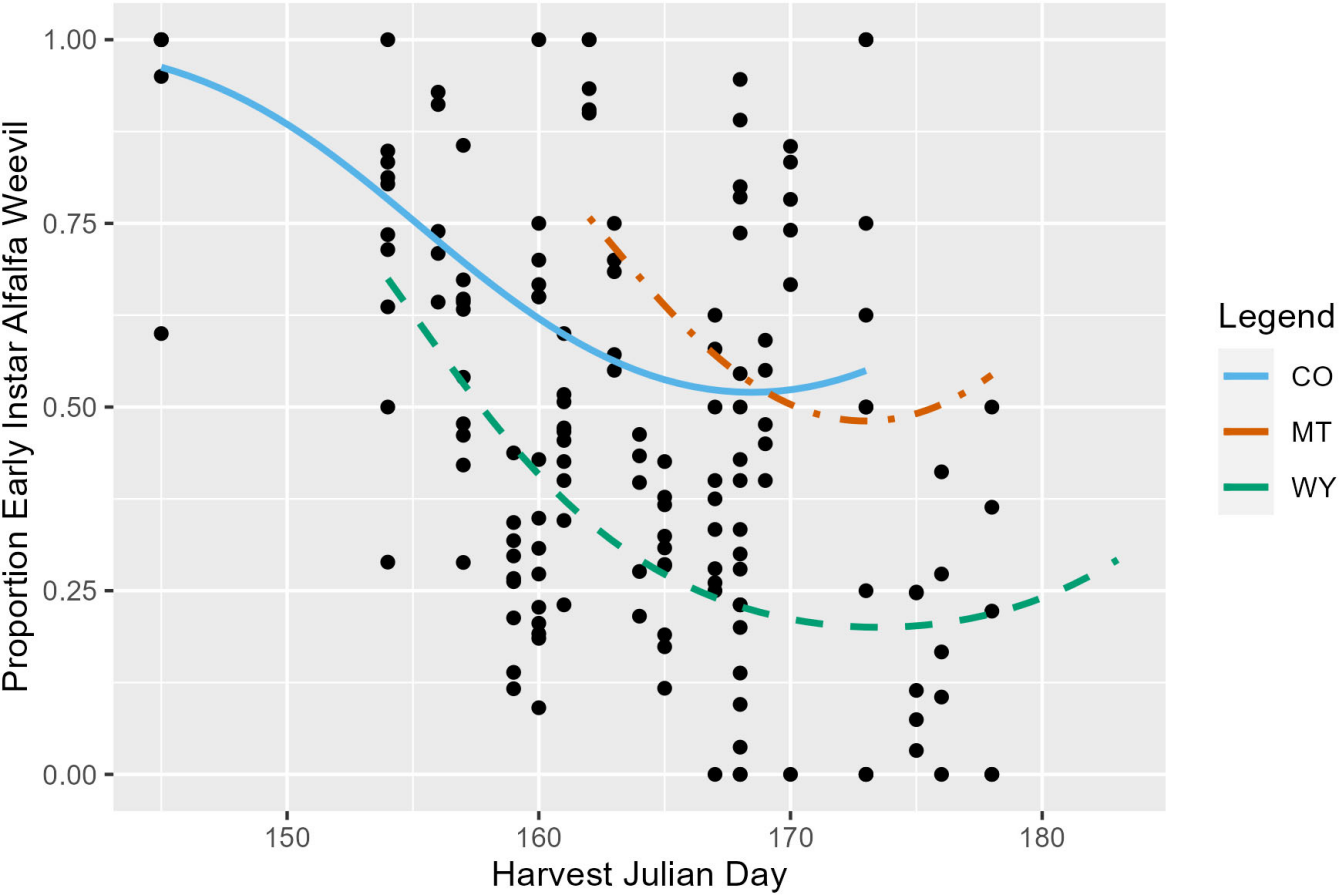
The proportion of all pre-harvest collected alfalfa weevil in a subplot that were at the first or second instar larvae stage plotted against harvest Julian day. Each point represents a single 0.09-m^2^ (1-ft^2^) subplot so there are multiple points per unique field. Model predictions from Colorado (solid blue), Montana (dot-dashed orange), and Wyoming (dashed green) are plotted.

Harvest Julian day, alfalfa stage, and state were all significant predictors of the pre-harvest proportion of the total number of alfalfa weevil (larvae, pupae, and adult) that were at the early (first and second) instar stage. On average, the proportion of alfalfa weevils that were early instar larvae decreased as harvest Julian day increased (*p* ≤ 0.001; **Appendix 1**; **Figure 2**) and as alfalfa plant stage increased (*p* ≤ 0.001; **Appendix 1**; **Supplementary Figure 4**). Additionally, each state had significantly different alfalfa weevil stage proportions, with Montana having, on average, the highest proportion of early instar alfalfa weevil and Wyoming, on average, having the lowest proportion (**Appendix 1**; CO-MT: *p* ≤ 0.001; CO-WY: *p* ≤ 0.001; MT-WY: *p* ≤ 0.001). Finally, there was a significant interaction between harvest Julian day and alfalfa stage (*p* = 0.004; **Appendix 1**). The impact of harvest Julian day on the proportion of early instar larvae is variable between different alfalfa plant stages. At most alfalfa plant stages, there is a negative relationship observed between harvest Julian day and the early instar alfalfa weevil proportion, but the strength of this negative relationship varies by alfalfa plant stages (**Supplementary Figure 5**).

### 3.3 Post-harvest alfalfa weevil

**Figure 3:**
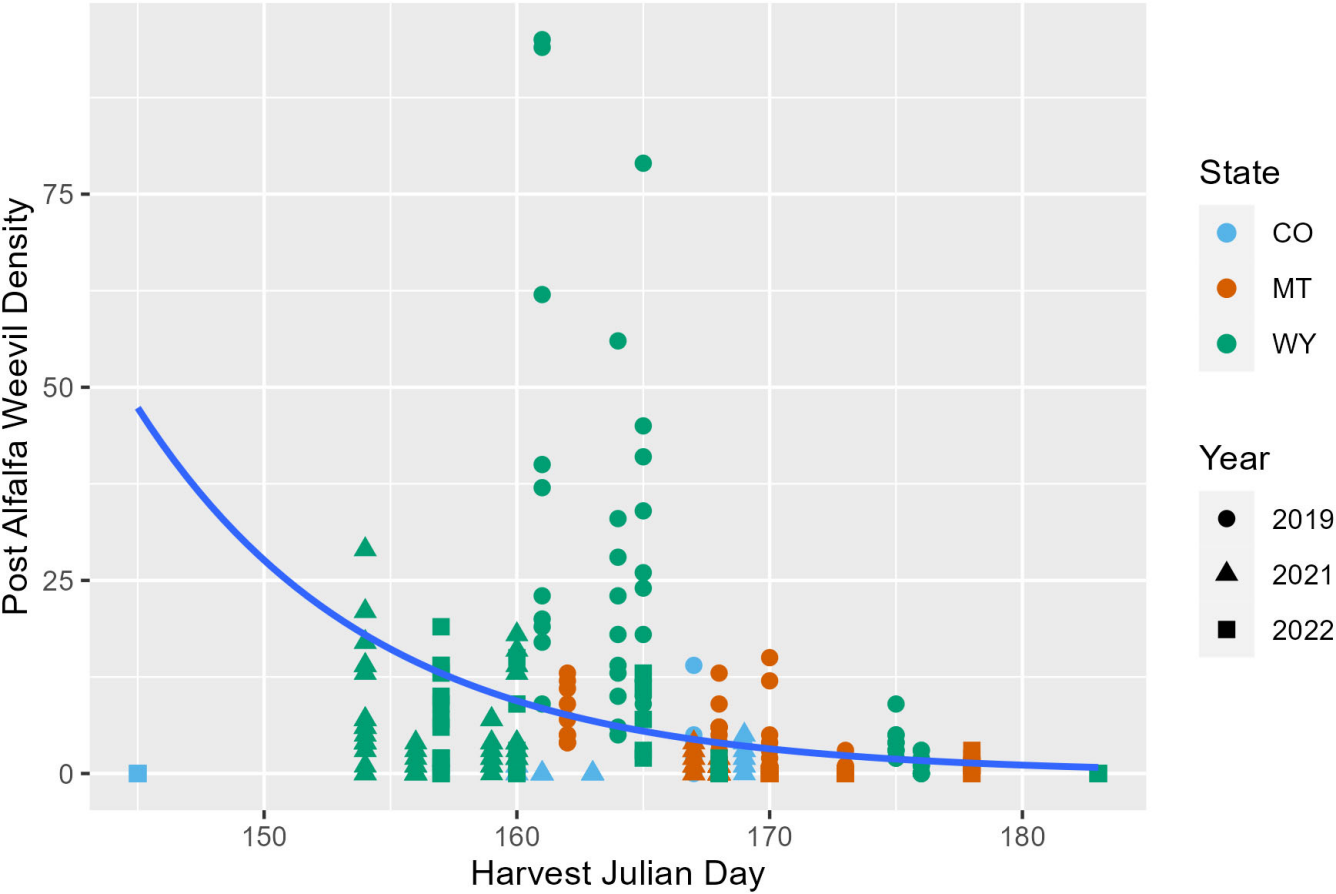
Post-harvest alfalfa weevil counts plotted against harvest Julian day with the average negative binomial regression line. Each point represents a single 0.09-m^2^ (1-ft^2^) subplot so there are multiple points per unique field. Point shape and color are representative of collection year (2019, circle; 2021, triangle; 2022, square) and state (CO, blue; MT, orange; WY, green).

**Figure 4:**
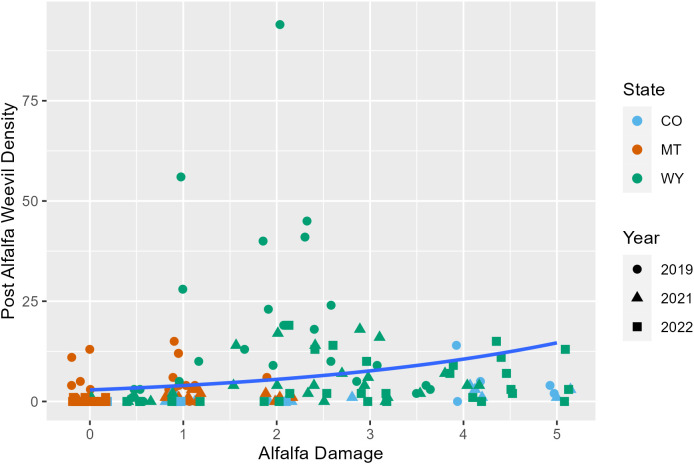
Post-harvest alfalfa weevil counts plotted against the alfalfa damage score pre-harvest with the average negative binomial regression line. Each point represents a single 0.09-m^2^ (1-ft^2^) subplot so there are multiple points per unique field. Point shape and color are representative of collection year (2019, circle; 2021, triangle; 2022, square) and state (CO, blue; MT, orange; WY, green).

Later harvest Julian days were associated with fewer alfalfa weevils in post-harvest samples (*p* = 0.02; **Appendix 1**; **Figure 3**; **Supplementary Figure 6**). This pattern was also observed in previous analyses examining producer management trends. Windrow status during post-harvest collections was also an important factor for post-harvest weevil densities. Fields that had windrows removed had approximately 0.5 fewer weevils per 0.09 m^2^ (1 ft^2^) than fields where windrows were still in place (**Appendix 1**; *p* = 0.007). Higher alfalfa damage scores observed pre-harvest were predictive of higher alfalfa weevil densities post-harvest (**Appendix 1**; *p* ≤ 0.001; **Figure 4**). Post-harvest alfalfa weevil densities also varied between states and collection years. More post-harvest alfalfa weevils were collected in Montana and Wyoming compared to Colorado, and 2019 had higher post-harvest alfalfa weevil densities compared to the other 2 years of collection (**Appendix 1**; CO-MT: *p* = 0.01; CO-WY: *p* ≤ 0.001; 2019–2021: *p* ≤ 0.001; 2019–2022: *p* ≤ 0.001). Pre-harvest alfalfa weevil egg count and early instar larvae proportions did not significantly impact post-harvest alfalfa weevil densities, but both contributed towards improving the overall AIC of our model.

### 3.4 Parasitoid activity

**Figure 5:**
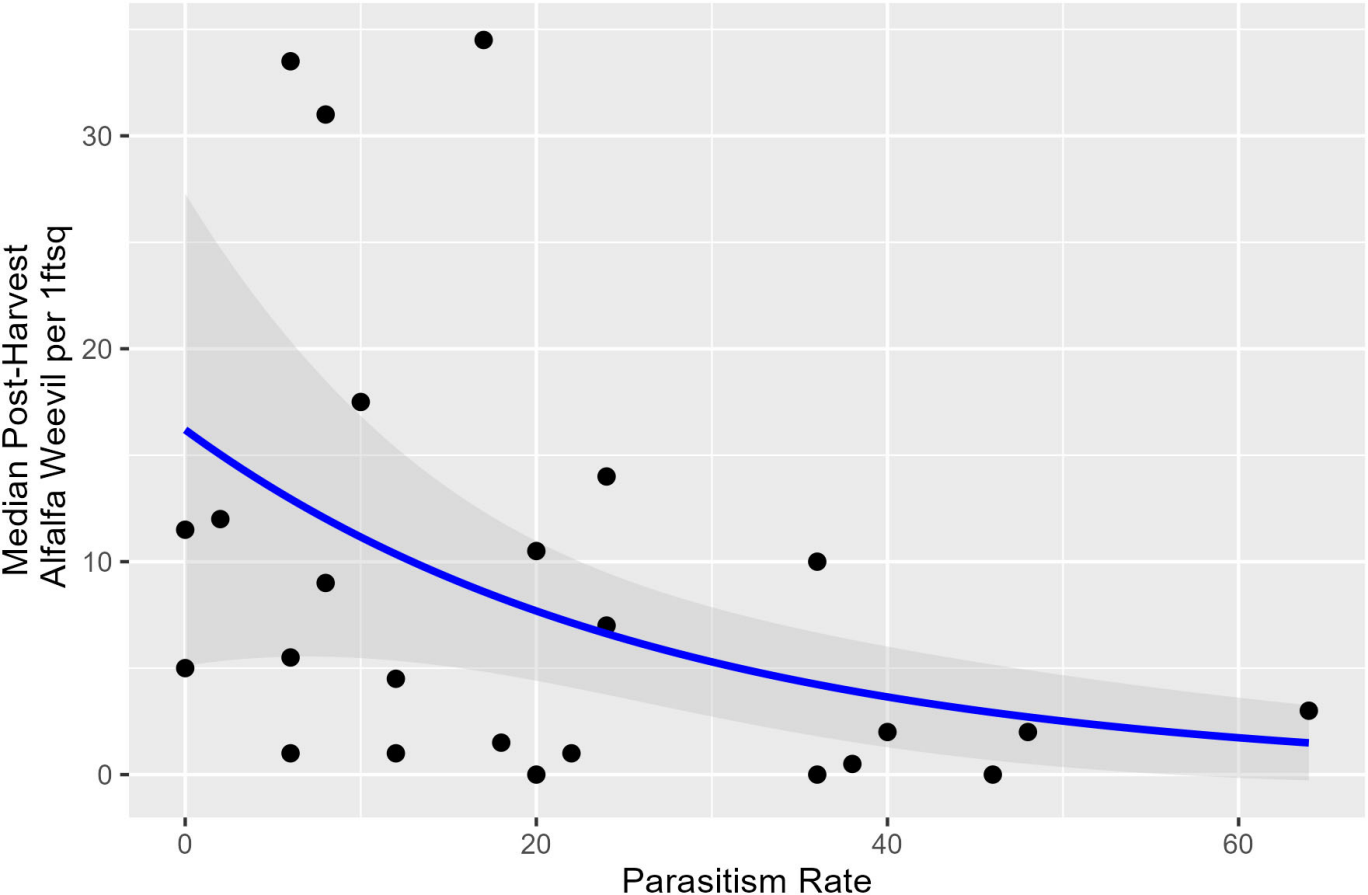
Post-harvest alfalfa weevil density plotted against pre-harvest percent parasitism rate with model predictions and its 95% confidence interval. Alfalfa weevil density is the median number based on all 0.09-m^2^ (1-ft^2^) subplots collected in each field, so each point represents a unique field.

Harvest Julian day, pre-harvest weevil density, and the proportion of early instar larvae were not associated with parasitism rates taken within a week prior to harvest. Overall, lower post-harvest weevil densities occurred when pre-harvest parasitism rates were higher (**Appendix 1**; **Figure 5**; *p* = 0.009).

## 4 Discussion

Initial harvest dates from fields sampled across the Intermountain West spanned a little over a month with the average day of first harvest in mid-June. Alfalfa stage at harvest varied widely from vegetative to flowering. Although we worked with a diversity of producers, our sampling is mostly representative of fields where the likelihood of insecticide use was low for various reasons, including potential producer perception that weevil pressure did not merit insecticide application as well as certified organic status. Moreover, producers in some regions harvested before sampling efforts began; thus, such samples were not included in this work. Shoemaker and Onstad (35) recommended harvesting between June 2 and 17 based on weather, weevil densities, and parasitoid densities for optimal alfalfa weevil control in central New York. GDDs are also a popular method of providing early harvest recommendations, but these are variable based on regions. A New York study found the optimal GDDs to be 340, whereas 507 was recommended in Michigan (9, 12). We did not have precise weather data for each field; thus, alfalfa weevil GDD models could not be obtained in this study. Early harvest was a common practice for our producer collaborators in the Intermountain West, with some harvesting a full week before historical early harvest recommendations. These shifts to early harvests could be due to various drivers: focusing on quality (i.e., higher nutritive value) rather than yield for particular markets, changes in climate impacting alfalfa and weevil growth rates, or use of newly developed alfalfa varieties (36, 37).

Alfalfa weevil densities, both pre- and post-harvest, decreased as harvest Julian day increased. This relationship could be a function of a few factors. Producers who have lower weevil pressure may harvest later because they have the ability to maximize yield without weevil damage trade-offs (11). In addition, alfalfa weevil populations are seasonal, being most active early in the growing season and then gradually decreasing from a variety of mortality factors (18, 38, 39). We found that the difference between predicted pre-harvest alfalfa weevil densities compared to predicted post-harvest alfalfa weevil densities were higher earlier in the season—highlighting that cutting a field later can result in smaller alfalfa weevil reductions and less impact. Casagrande and Stehr (9) previously found a similar trend, with the reduction in ovipositing alfalfa weevils being less at later harvests.

Early harvest is thought to be an effective strategy because alfalfa cutting happens before alfalfa weevil damage takes place while also targeting early instar alfalfa weevil larvae that are vulnerable to mortality (40, 41). Our finding that later harvest Julian days and alfalfa plant stages are associated with a lower proportion of early instar alfalfa weevil supports the notion that earlier harvests based on either date or host plant development will target first and second instar alfalfa weevils. Alfalfa weevil stages differed between states in our study, with Wyoming having the lowest proportion of early instar alfalfa weevil and Montana having the highest. This could be influenced, in part, by the latitudinal differences between states and associated climates found throughout the Intermountain West. For example, De Frenne and colleagues (42) showed the importance of latitudinal gradients in explaining variation in plant traits, which could result from differences in temperature along such a gradient, but also potentially other environmental drivers.

Alfalfa weevil post-harvest densities were impacted by a few different variables that help us better understand the role of harvest timing and alfalfa management. We found that higher levels of alfalfa weevil damage were positively associated with higher post-harvest weevil densities. Alfalfa weevil density and alfalfa weevil damage have been shown to be positively correlated in previous studies (21, 23). Since harvest does not completely eliminate all alfalfa weevils in the field, the association between damage and post-harvest weevil density is potentially due to increased alfalfa weevil activity corresponding to higher weevil densities remaining in the field after harvest (9, 43). Post-harvest weevil densities are a major concern of producers who are critical of the efficacy of early harvest, aligned with concerns that weevils will negatively impact their second cutting.

In our study, the lower rates of alfalfa weevil densities on fields without windrows may be associated with the potential of windrows to serve as refuges for alfalfa weevil in harvested fields (26). If windrows are not removed from a field quickly, alfalfa weevil can potentially cause damage to regrowing alfalfa (44). Blodgett and Lenssen (26) showed that as the windrows dried, alfalfa weevil likely died or moved onto the field; removing windrows in a timely manner could also remove the weevils in the windrows, preventing them from moving into the field. Our work supports the timely removal of windrows to decrease post-harvest alfalfa weevil densities.

There were no clear associations between harvest Julian day and the parasitism rate of alfalfa weevil by *B. curculionis* in our study. However, harvest Julian day and weevil densities were associated in our study (**Figure 1**), and others have documented a density-dependent relationship between alfalfa weevil and *B. curculionis* parasitism rates (19, 45). Our observation that a lower parasitism rate measured pre-harvest was associated with higher post-harvest alfalfa weevil densities agrees with other findings. Density-dependent relationships are common in host–parasitoid interactions (46, 47). *Bathyplectes curculionis* are efficient at finding and parasitizing alfalfa weevil hosts. When densities are low, they maintain high parasitism rates, but their rate of increase is slower than the alfalfa weevil; thus, high levels of parasitism are not maintained. Parasitism rates in our study were estimated within a week of the harvest Julian day. Measurement of parasitism rates over a longer time period, including the same day as harvest timing and careful estimates post-harvest when densities are lowered, could offer further clarity on how best to integrate harvest timing recommendations with biological control. Nonetheless, the use of early harvest rather than broad-spectrum insecticides is likely advantageous for beneficial insects, including parasitoids.

## 5 Conclusion

Across the Intermountain West, the initial alfalfa harvest, as decided by producers, spans over 1 month and a variety of alfalfa plant stages. In general, we see greater predicted differences between pre- and post-harvest alfalfa densities in producer fields that are harvested earlier compared to fields with later harvests. Beneficial management practices that producers would likely consider include harvesting before substantial pre-harvest damage is present and removing windrows as quickly as possible to lower post-harvest weevil densities. The optimal harvest timing to maximize *B. curculionis* impact remains unclear. This information would be beneficial to future alfalfa weevil management given that *B. curculionis* appear efficient at parasitizing alfalfa weevil at low densities typical of those observed post-harvest.

## Data availability statement

The raw data supporting the conclusions of this article will be made available by the authors, without undue reservation.

## Ethics statement

The manuscript presents research on animals that do not require ethical approval for their study.

## Author contributions

JH: Conceptualization, Data curation, Formal Analysis, Investigation, Methodology, Supervision, Visualization, Writing – original draft, Writing – review & editing. TR: Conceptualization, Funding acquisition, Investigation, Methodology, Project administration, Writing – review & editing. DC: Investigation, Methodology, Supervision, Writing – review & editing. FP: Funding acquisition, Methodology, Project administration, Writing – review & editing. RJ: Conceptualization, Funding acquisition, Investigation, Methodology, Project administration, Resources, Supervision, Writing – review & editing.
