# Supplementary material for: On-farm harvest timing effects on alfalfa weevil across the Intermountain West region of the United States
Source: Front Insect Sci. 2024 Apr 23;4:1324044. doi: 10.3389/finsc.2024.1324044 (PMC11075068; doi:10.3389/finsc.2024.1324044)
Supplement: Supplementary file 1 [file DataSheet_1.pdf]

frontiers

Supplementary Material

Supplementary Table 1. Description of geographic regions that were used to categorize field sites for analysis.

	Description of Field Site Regions
Colorado	In rural areas north of Fort Collins, CO
	Near urban areas south of Fort Collins, CO
	Near urban areas south of Loveland, CO
Montana	East of the Yellowstone River
	West of the Yellowstone River
	North of the Missouri River
Wyoming	Agricultural area near Wheatland, WY
	Agricultural area near Torrington, WY
	Fields near Chugwater, WY in semi-natural environments

Supplementary Table 2. Predicted pre- and post-harvest alfalfa weevil densities and their 95% confidence intervals (CI) from both negative binomial generalized linear models that examined producer management trends.

Harvest Julian	Pre-harvest Density (95% CI)	Post-harvest Density (95% CI)
154	21.24 (14.69, 30.7)	253.41 (176.98, 362.85)
155	18.82 (13.33, 26.57)	223.93 (159.94, 313.51)
156	16.68 (12.08, 23.01)	197.87 (144.45, 271.06)
157	14.78 (10.95, 19.95)	174.85 (130.35, 234.54)
158	13.09 (9.91, 17.31)	154.51 (117.52, 203.14)
159	11.6 (8.95, 15.04)	136.53 (105.81, 176.17)
160	10.28 (8.08, 13.09)	120.65 (95.13, 153)
161	9.11 (7.27, 11.42)	106.61 (85.37, 133.13)
162	8.07 (6.53, 9.98)	94.21 (76.43, 116.11)
163	7.15 (5.85, 8.75)	83.25 (68.25, 101.54)
164	6.34 (5.22, 7.7)	73.56 (60.74, 89.08)
165	5.62 (4.64, 6.81)	65 (53.87, 78.43)
166	4.98 (4.1, 6.04)	57.44 (47.6, 69.31)
167	4.41 (3.62, 5.38)	50.76 (41.9, 61.48)
168	3.91 (3.18, 4.81)	44.85 (36.76, 54.73)
169	3.46 (2.78, 4.31)	39.63 (32.14, 48.87)
170	3.07 (2.43, 3.88)	35.02 (28.03, 43.76)
171	2.72 (2.12, 3.5)	30.95 (24.38, 39.28)
172	2.41 (1.84, 3.16)	27.35 (21.18, 35.31)
173	2.14 (1.6, 2.86)	24.16 (18.36, 31.8)
174	1.89 (1.38, 2.59)	21.35 (15.9, 28.67)
175	1.68 (1.2, 2.34)	18.87 (13.76, 25.87)
176	1.49 (1.04, 2.13)	16.67 (11.9, 23.37)
177	1.32 (0.9, 1.93)	14.73 (10.28, 21.12)
178	1.17 (0.78, 1.75)	13.02 (8.88, 19.1)
179	1.03 (0.67, 1.59)	11.5 (7.66, 17.28)
180	0.92 (0.58, 1.45)	10.17 (6.61, 15.63)
181	0.81 (0.5, 1.31)	8.98 (5.7, 14.15)
182	0.72 (0.43, 1.2)	7.94 (4.92, 12.82)
183	0.64 (0.37, 1.09)	7.01 (4.24, 11.61)

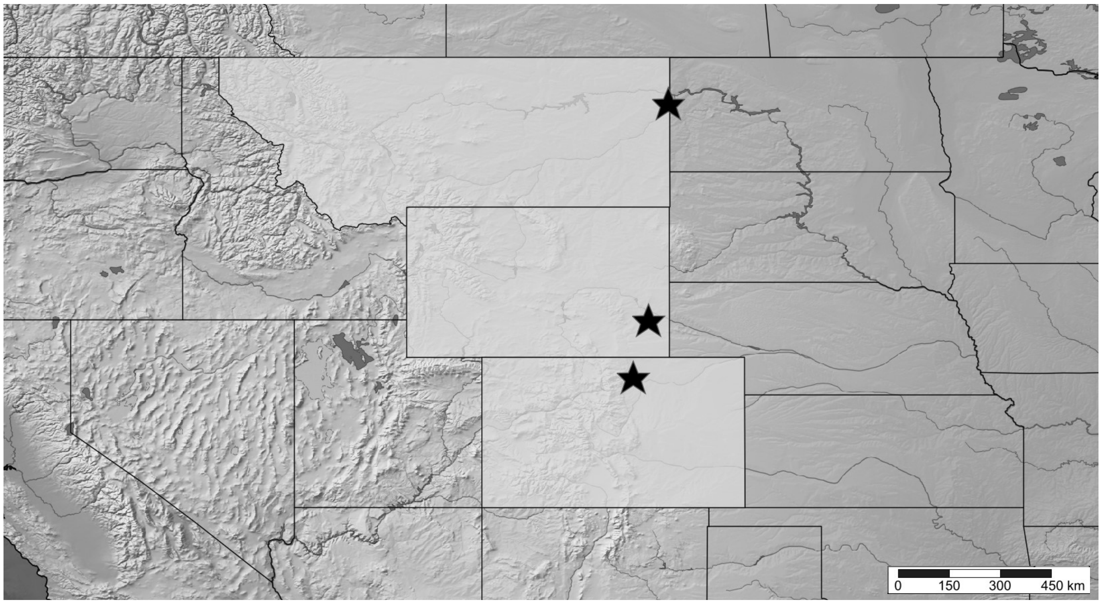

Supplementary Figure 1. Stars illustrate the general location of our field sites in eastern Montana (and to a lesser extent western North Dakota), southeastern Wyoming, and northern Colorado. Created using SimpleMappr.

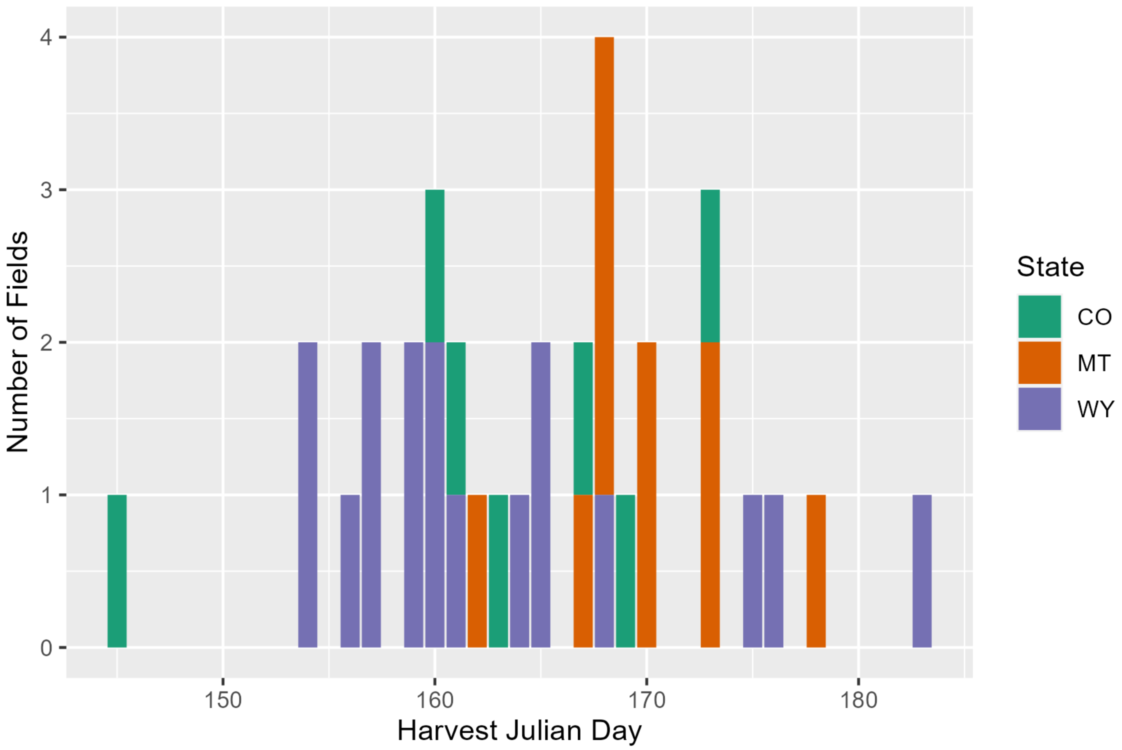

Supplementary Figure 2. Julian day of harvest for each field sampled over 2019, 2021, and 2022.

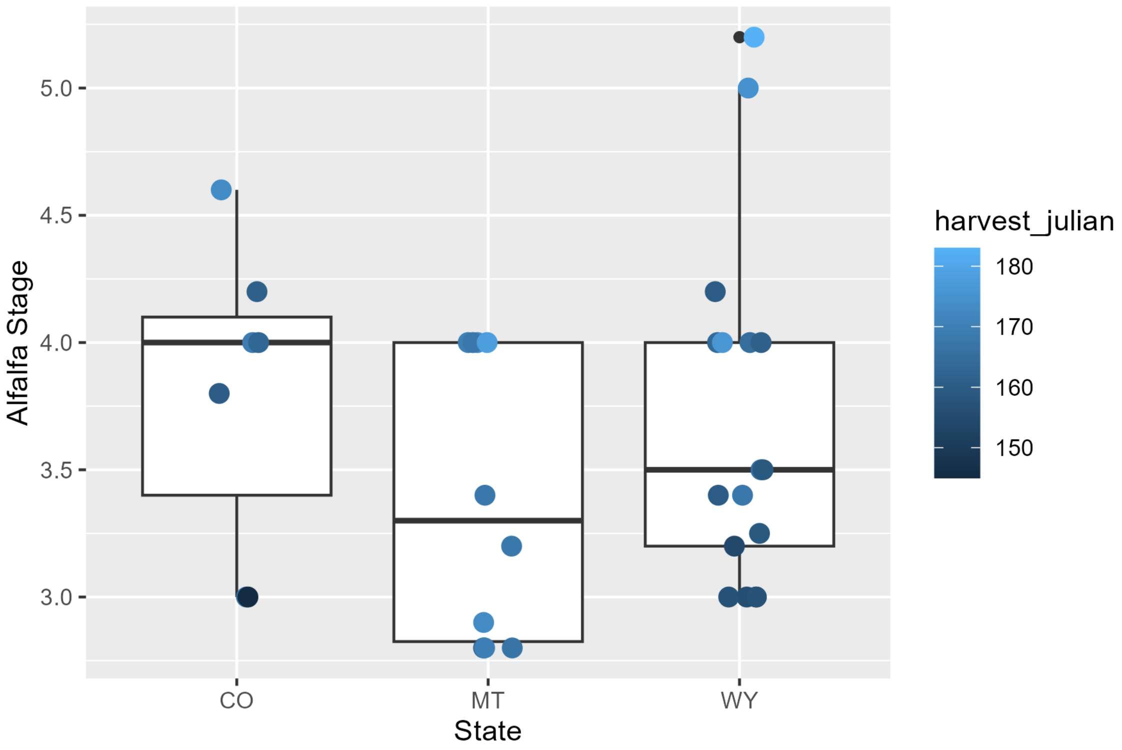

Supplementary Figure 3. Alfalfa plant stage in each state before harvest. Points are shaded based on their harvest Julian date (harvest_julian). Each point represents a single field.

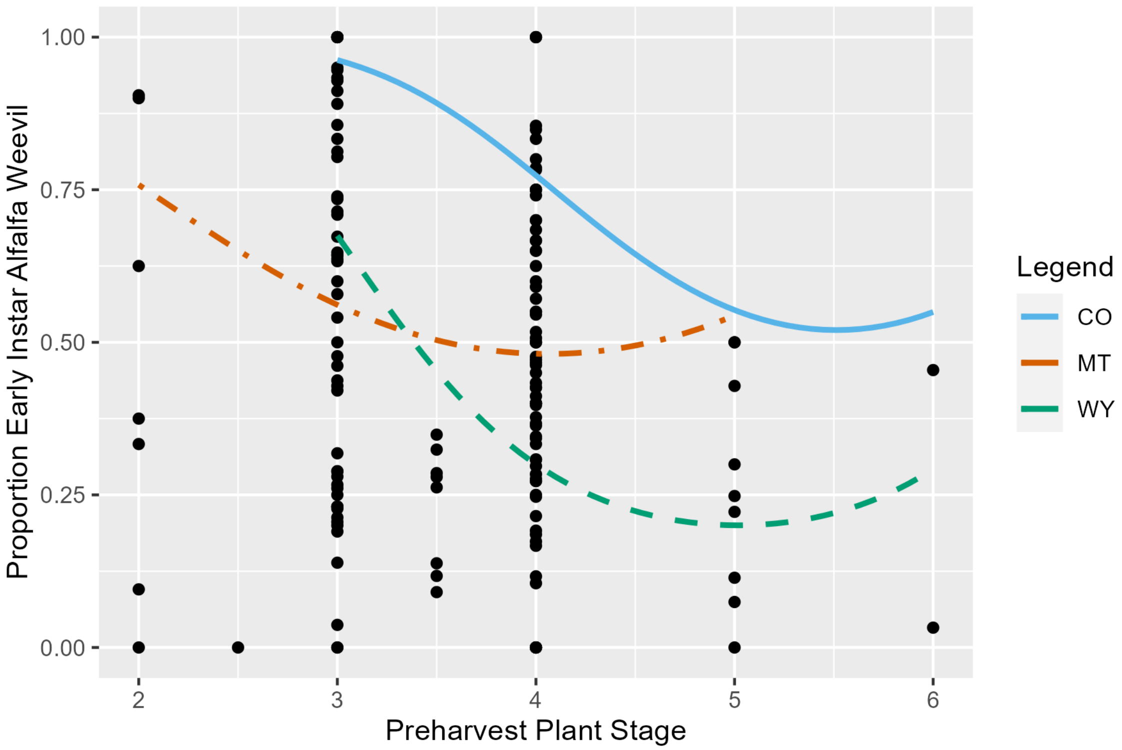

Supplementary Figure 4. The proportion of all pre-harvest collected alfalfa weevil in a subplot that were at the 1st or 2nd instar larvae stage plotted against the pre-harvest alfalfa plant stage. Each point represents a single 0.09 m2 (1ft2) subplot so there are multiple per unique field. Model predictions from Colorado (solid blue), Montana (dot-dashed orange), and Wyoming (dashed green) are plotted.

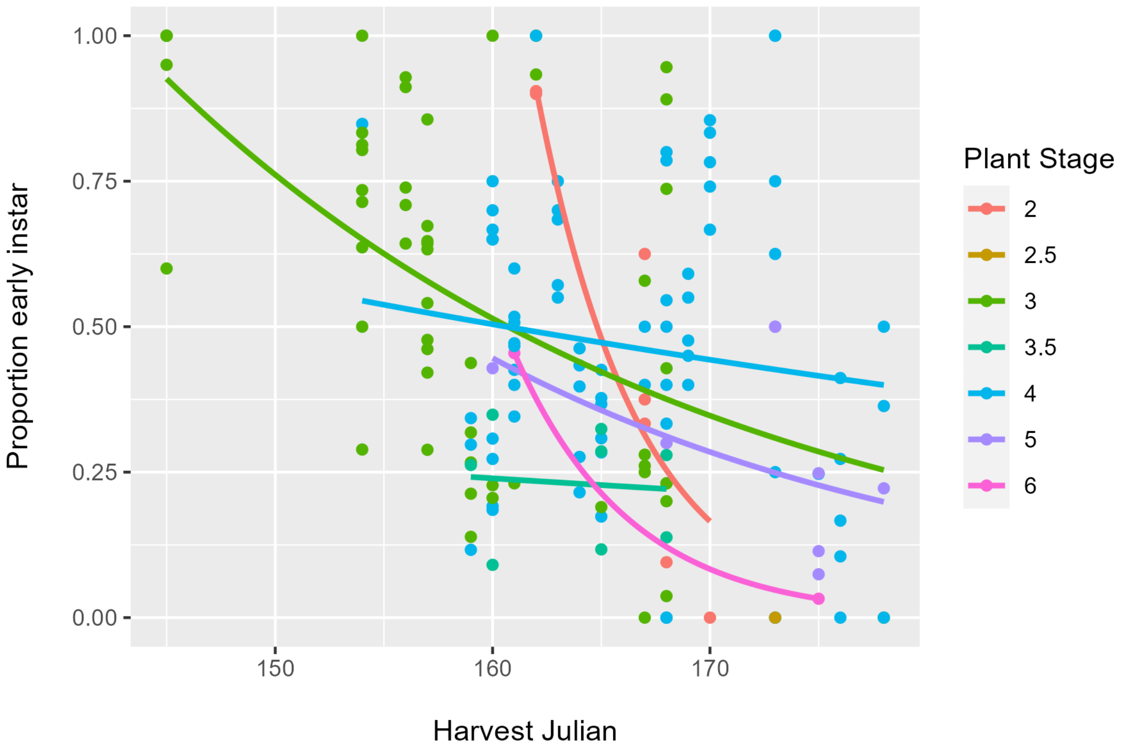

Supplementary Figure 5. The proportion of all pre-harvest collected alfalfa weevil in a subplot that were at the 1st or 2nd instar larvae stage plotted against harvest Julian day with the average quasibinomial regression line for each recorded alfalfa plant stage. Alfalfa weevil density is the median number based on all 0.09 m^2 (1ft^2) subplots collected in each field, so each point is representative of a unique field.

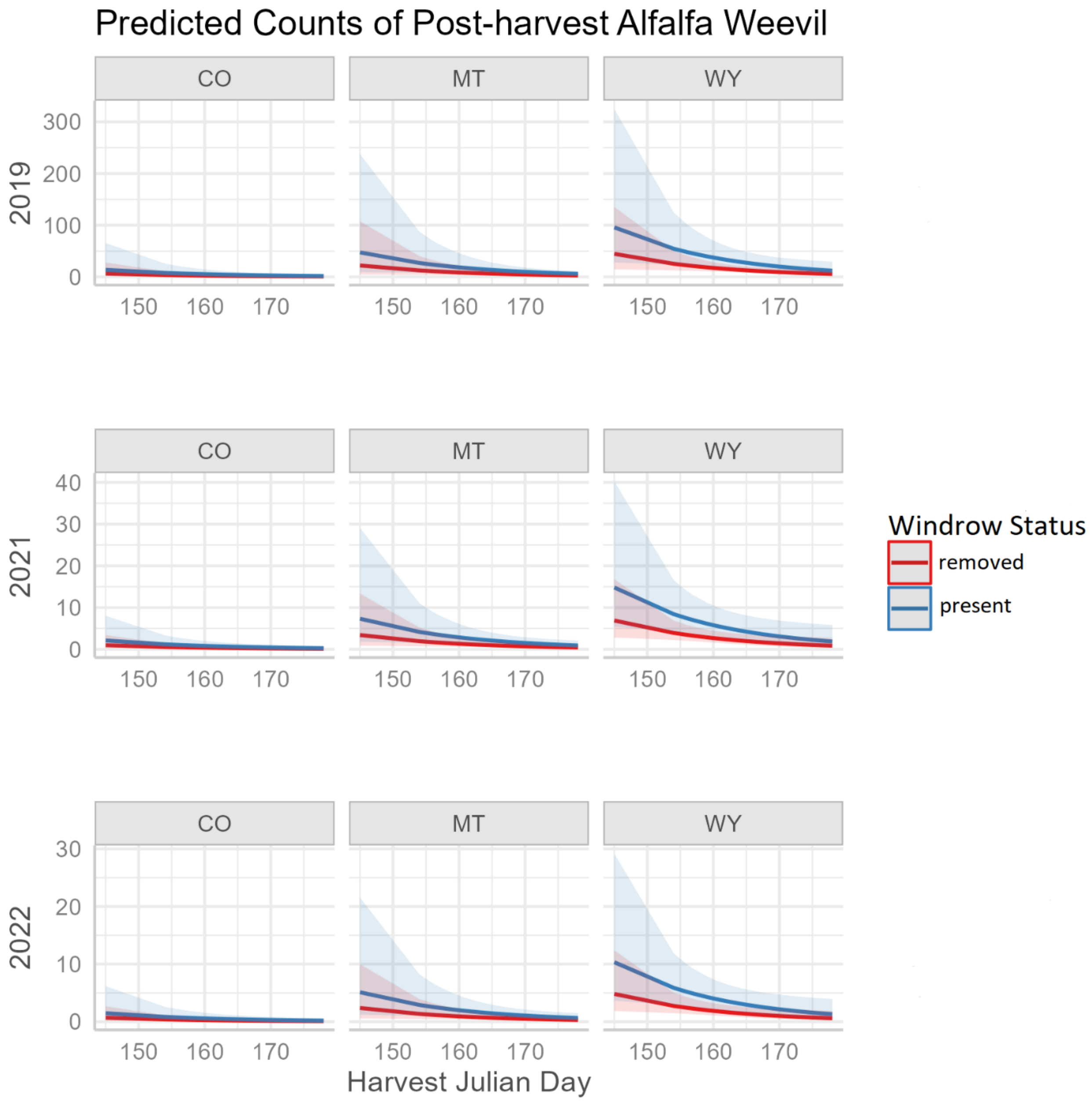

Supplementary Figure 5. Post-harvest weevil density predictions across harvest Julian day. Predictions are derived from the final generalized linear mixed model of post-harvest alfalfa weevil. Predictions are based on average pre-harvest alfalfa damage, alfalfa weevil egg count and early instar larvae proportions.

Management Trends Model Summaries

Harvest Julian Day ~ State

	Df	Sum Sq	Mean Sq	F value	Pr(>F)
State	2	322.3	161.15	2.875	0.0715
Residuals	31	1737.6	56.05		

Alfalfa Plante Stage ~ State

	Df	Sum Sq	Mean Sq	F value	Pr(>F)
State	2	0.828	0.4141	1.053	0.361
Residuals	31	12.188	0.3932		

Weevil Density Pre-harvest ~ Harvest Julian Day

	Estimate	Std. Error	z value	Pr(>|z|)	
(Intercept)	24.58337	2.23798	10.985	<2e-16	***
harvest_julian	-0.12369	0.01351	-9.154	<2e-16	***

Weevil Density Post-harvest ~ Harvest Julian Day

	Estimate	Std. Error	z value	Pr(>|z|)	
(Intercept)	21.67500	2.35529	9.203	<2e-16	***
harvest_julian	-0.12090	0.01424	-8.489	<2e-16	***

Alfalfa Weevil Instar Proportion Model Summary

	Estimate	Std. Error	t value	Pr(>|t|)	
(Intercept)	54.98666	11.77786	4.669	0.00000671	***
StateMT	0.97395	0.26704	3.647	0.000366	***
StateWY	-0.91583	0.22697	-4.035	0.00008695	***
harvest_julian	-0.33905	0.07142	-4.747	0.00000480	***
PlantStage	-8.64755	3.09202	-2.797	0.005844	**
harvest_julian:PlantStage	0.05371	0.01862	2.885	0.004492	**

Post-Harvest Alfalfa Weevil Model Summary

	Estimate	Std. Error	z value	Pr(>|z|)	
(Intercept)	10.628175	4.843888	2.194	0.02823	*
prop.early.pre	0.167915	0.575121	0.292	0.77031	
Year2021	-1.867015	0.283909	-6.576	4.83e-11	***
Year2022	-2.227586	0.300996	-7.401	1.35e-13	***
harvest_julian	-0.062733	0.027389	-2.290	0.02199	*
Damage	0.466840	0.103760	4.499	6.82e-06	***
`total_eggs_per25stems_-1`	0.016109	0.009093	1.772	0.07648	
windremoval_2rmv	-0.763639	0.281942	-2.709	0.00676	**
StateMT	1.244207	0.494502	2.516	0.01187	*
StateWY	1.948146	0.423226	4.603	4.16e-06	***

Parasitism Activity Model Summaries

Parasitism Rate Model

	Estimate	Std. Error	t value	Pr(>|t|)
(Intercept)	4.571785	8.595080	0.532	0.601
Total_AW_pre	-0.005232	0.003923	-1.334	0.197
proportion_early	-0.605455	1.005139	-0.602	0.554
harvest_julian	-0.031681	0.049165	-0.644	0.527

Weevil Density Post-harvest ~ Parasitism Rate

	Estimate	Std. Error	t value	Pr(>|t|)	
(Intercept)	2.78460	0.34937	7.970	0.0000000457	***
parasitism	-0.03727	0.01308	-2.849	0.00909	**

Supplementary Appendix 1: Summary outputs for all models referenced in results section.
